# Mathematical optimization in classification and regression trees

**DOI:** 10.1007/s11750-021-00594-1

**Published:** 2021-03-17

**Authors:** Emilio Carrizosa, Cristina Molero-Río, Dolores Romero Morales

**Affiliations:** 1 Instituto de Matemáticas de la Universidad de Sevilla, Seville, Spain; 2 Department of Economics, Copenhagen Business School, Frederiksberg, Denmark

**Keywords:** Classification and regression trees, Tree ensembles, Mixed-integer linear optimization, Continuous nonlinear optimization, Sparsity, Explainability, 90C11, 90C30, 62-07

## Abstract

Classification and regression trees, as well as their variants, are off-the-shelf methods in Machine Learning. In this paper, we review recent contributions within the Continuous Optimization and the Mixed-Integer Linear Optimization paradigms to develop novel formulations in this research area. We compare those in terms of the nature of the decision variables and the constraints required, as well as the optimization algorithms proposed. We illustrate how these powerful formulations enhance the flexibility of tree models, being better suited to incorporate desirable properties such as cost-sensitivity, explainability, and fairness, and to deal with complex data, such as functional data.

## Introduction

Extracting knowledge from data is a crucial task in Statistics and Machine Learning, and is at the core of many fields, such as Biomedicine (Jakaitiene et al. 2016; Pardalos et al. 2007); Business Analytics (Martens et al. 2007; Van Vlasselaer et al. 2017), Computational Optimization (Khalil et al. 2016; Lodi and Zarpellon 2017), Criminal Justice (Ridgeway 2013; Zeng et al. 2017), Cybersecurity (Kaloudi and Li 2020; Martínez Torres et al. 2019), Health Care (Bertsimas et al. 2016; Souillard-Mandar et al. 2016), Policy Making (Kleinberg et al. 2018; Wager and Athey 2018), Process Monitoring (Apsemidis et al. 2020), Regulatory Benchmarking (Benítez-Peña et al. 2020a; Esteve et al. 2020). Mathematical Optimization plays an important role in building such models (Bertsimas and Shioda 2007; Fang et al. 2013; Fountoulakis and Gondzio 2016; Goodfellow et al. 2016), in interpreting their output (Carrizosa et al. 2020b; Dash et al. 2018; Rudin and Ertekin 2018; Ustun and Rudin 2016) or visualizing it (Carrizosa et al. 2017, 2018a, b, 2020a, c). See Bottou et al. (2018), Gambella et al. (2020), Liberti (2020) for surveys reviewing the use of Mathematical Optimization in Machine Learning, and Carrizosa and Romero Morales (2013), Duarte Silva (2017), Palagi (2019), and Piccialli and Sciandrone (2018) for surveys focusing on specific methodologies.

**Fig. 1:**
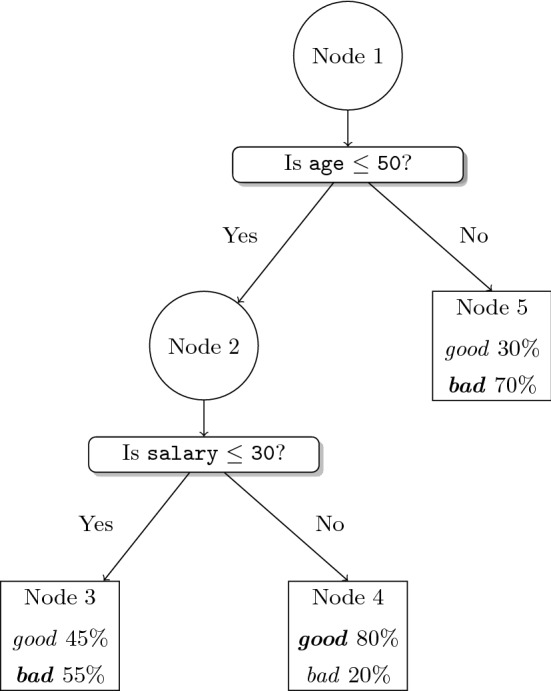
A tree model $\mathcal {T}$ to predict the *good* payers class vs the *bad* payers class, with $\tau _B = \{\text{ Node } \text{1},\text{ Node } \text{2}\}$ and $\tau _N = \{\text{ Node } \text{3},\text{ Node } \text{4},\text{ Node } \text{5}\}$; orthogonal cuts age $\le 50$ and salary $\le 30$; and prediction *good* for Node 4 and *bad* for Node 3 and Node 5

Classification and regression trees (Loh 2014) are state-of-the-art methods based on recursive partitioning (Hastie et al. 2009). They are conceptually simple, show excellent learning performance, are computationally cheap, and routines and packages to train them are available in popular languages such as Python and R, and are also appealing in terms of interpretability (Freitas 2014; Hu et al. 2019; Lin et al. 2020; Meinshausen 2010) because of their rule-based nature. This makes them popular in many applications, including, for instance, a credit scoring exercise for granting a loan, described in what follows for illustration purposes. There, we have a dataset of individuals characterized by demographic and financial predictor variables, among others, and, with this information, the model predicts whether customers will be *good* or *bad* payers. In Fig. 1, we have a stylized credit scoring tree that will help us visualize some of the concepts reviewed in this paper.

To construct a tree model, say $\mathcal {T}$, one has at hand a training sample $\mathcal {I}=\left\{ \left( \varvec{x}_i,y_i\right) \right\} _{ 1\le i \le N}$, with $\varvec{x}_i\in \mathbb {R}^p$ the vector of predictor variables and $y_i$ the response variable of individual *i*. Note that wlog we assume categorical variables have been modeled through dummy ones, and thus replaced by a set of binary variables indicating the presence/absence of each category. The nature of the response variable depends on whether we are dealing with a classification or a regression task. For classification, $y_i \in \{ 1,\ldots , K\}$ is the class label associated with individual *i*, while for regression, $y_i \in \mathbb {R}$ is a continuous measurement.

The main goal of a classification and regression tree $\mathcal {T}$ is to predict, as accurately as possible, the response variable *y* using the predictor variables $\varvec{x}$. On the top of this primary goal, other important characteristics may need to be considered, such as, e.g., cost-sensitivity constraints to protect risk groups (Kao and Tang 2014; Turney 1995), fairness of the method avoiding the discrimination of groups that share sensitive features such as gender and race (Aghaei et al. 2019; Miron et al. 2020; Romei and Ruggieri 2014; Zafar et al. 2017), and explainability properties, e.g., sparsity and local interpretability of the tree model (Lundberg et al. 2020; Lundberg and Lee 2017; Molnar et al. 2020; Ribeiro et al. 2016).

A tree model $\mathcal {T}$ consists of a tree decision structure and a prediction structure. The tree decision structure of $\mathcal {T}$ is defined by two elements, namely, the topology of the tree, i.e., the branch nodes $\tau _B$ and the leaf nodes $\tau _L$, as well as the arcs between them, and the splitting rules applied at the branch nodes. The prediction structure is defined by the (statistical) prediction models attached to the leaf nodes. To illustrate these concepts, consider the topology of the tree model in Fig. 1, which consists of two branch nodes, Node 1 and Node 2, and three leaf nodes, Node 3, Node 4, and Node 5. This is a binary tree, since each branch node has two children. The root node is where all individuals of $\mathcal {I}$ start. These individuals move along the tree according to the queries asked at the branch nodes. In this way, and after partitioning the training sample $\mathcal {I}$ successively, each individual ends up reaching exactly one leaf node, yielding $\mathcal {I} = \cup _{t \in \tau _L} \mathcal {I}_t$ with $\mathcal {I}_t \cap \mathcal {I}_{t'} =\emptyset$ for $t\not = t'$. In terms of splitting rules, the query asked in this example at the root node is whether predictor variable age is below 30, while at Node 2, we ask whether salary is below 50. The purpose of this splitting process is to ensure that individuals in the same leaf node follow the same pattern (i.e., they are from the same class or their response variable can be accurately predicted by a unique model, such as, for instance, a linear or a logistic model) and such pattern is expected to be also present at new individuals falling inside this leaf node. The prediction in leaf node *t* is chosen fitting a model to the subsample $\mathcal {I}_t$. In Fig. 1, we can see that Node 4 predicts individuals as *good* payers, since this is the most frequent class (in bold font) in Node 4, while, following a similar argument, the other two leaf nodes predict as *bad* payers.

Once the tree model is built, the prediction of future data is done in a deterministic way. Given a new observation $\varvec{x}_\mathrm{new}$, starting from the root node, and applying the queries at the branch nodes, it will end up in a leaf node, say $t(\varvec{x}_\mathrm{new}) \in \tau _L$. The prediction made for $\varvec{x}_\mathrm{new}$ is that associated with leaf node $t(\varvec{x}_\mathrm{new})$. In our example, a new individual of age 43 and salary 28 would end up in Node 3, and therefore, it would be predicted as a *bad* payer.

Mathematical Optimization is present at the three elements that define a tree model, namely, topology design of the tree, branching, and prediction. First, we face the problem of designing the topology of the tree. This network design problem is often avoided, by, e.g., choosing a binary tree of depth *D*, for a given value of *D*. To make this decision more data-dependent, a larger tree is built and pruned afterward, collapsing existing leaf nodes into new ones containing more individuals. See, e.g., Sherali et al. (2009) for structural properties of the optimization problem associated with the pruning step. In this way, one obtains a more parsimonious tree, which is expected to perform better for future individuals. Second, we have to decide the splitting rules at each branch node. It is common to see trees implementing splitting rules that correspond to so-called orthogonal cuts, i.e., queries involving a single predictor variable, say $x_j \le \mu$. The choice of the predictor variable $x_j$ and the threshold value $\mu$ can be modeled with 0-1 decision variables. However, it is common to see enumerative procedures being applied independently to each of the branch nodes, thus solving the problem locally and not globally. Although orthogonal cuts are popular (easy to build and to interpret), higher efficiency can be achieved with more sophisticated splitting rules, such as, e.g., linear oblique cuts, i.e., queries of the form $\sum _{j=1}^p a_j x_j \le \mu$. See, e.g., Street (2005) for the optimization of oblique cuts. Third, and last, we need to decide how predictions are made at each leaf node $t \in \tau _L$. This boils down to solving an optimization problem for each $t \in \tau _L$, the shape of which depends on the nature of the response variable. For instance, in a regression tree, predictions can be made with a linear model obtained through an Ordinary Least Squares model. See, e.g., Demirović and Stuckey (2020) for the optimization of other criteria to measure the quality of prediction.

Because of the availability of more powerful hardware and the dramatic advances in optimization solvers over the last decades, there has been an increased interest by the Mathematical Optimization community to develop novel approaches to build classification and regression trees. In this paper, we review recent contributions within the Continuous Optimization and the Mixed-Integer Linear Optimization (MILO) paradigms, which both have shown good accuracies compared to the traditional heuristic approaches. We compare the Continuous Optimization and the MILO models in terms of the nature of the decision variables and the constraints, as well as other characteristics related to extracting explainability results to aid Data-Driven Decision-Making. Having these powerful formulations enhances the flexibility of tree models to incorporate desirable properties in data science models, stemming from different fields of application, compared to the greedy heuristic approaches.

The remainder of the paper is organized as follows. In Sect. 2, we briefly go through the simplest (greedy heuristic) approaches to construct classification and regression trees, as well as extensions such as Random Forests, to understand how the one-shot optimization of the decisions across the whole tree is overcome. In Sect. 3, we review the Continuous Optimization and the MILO paradigms to build optimal decision trees, and how they compare against each other. In Sect. 4, we describe recent progress of the mathematical optimization community to incorporate important desirable model properties in tree models, and pose new lines of research in this burgeoning area. Finally, Sect. 5 concludes the paper.

## Greedy classification and regression trees

Throughout this section, we discuss optimization approaches that focus on the design of specific components of the tree model (Aglin et al. 2020; Bennett and Blue 1996; Nijssen and Fromont 2010; Savickỳ et al. 2020). Section 2.1 reviews the basic steps of greedy heuristics to build classification and regression trees with orthogonal cuts. We continue with generalizations that aim at enhancing accuracy. In Sect. 2.2, we discuss tree models with more complex cuts, while Sect. 2.3 is devoted to models that combine a collection of trees. Finally, Sect. 2.4 challenges the greedy approach when the user needs to control other objectives in addition to the accuracy of the tree model.

### Building a tree model with orthogonal cuts

**Fig. 2:**
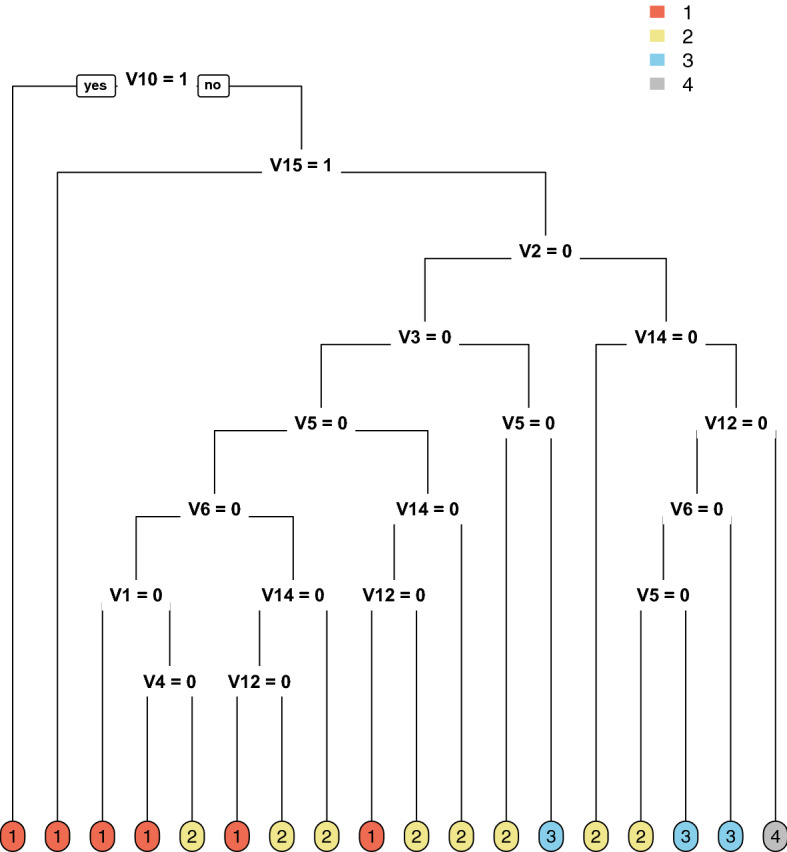
Illustration of CART for carevaluations obtained with the R package rpart (Therneau et al. 2015). There are 16 leaf nodes, predicting one of the four classes, namely *unacceptable* (1), *acceptable* (2), *good* (3), or *very good* (4). The classification accuracy provided by this model is $88.1\%$, while $71.3\%$ of the predictor variables are used across the tree

Since constructing optimal binary classification and regression trees is known to be an NP-complete problem (Hyafil and Rivest 1976), early research traditionally focused on the design of greedy heuristic procedures (Yang et al. 2017) that require a low computational effort to build tree models with just orthogonal cuts. These are recursive partitioning methods that build the tree model in a forward stepwise search implementing orthogonal cuts, yielding binary trees, e.g., CART (Breiman et al. 1984) and QUEST (Loh and Shih 1997), or nonbinary trees, a.k.a. multi-way trees (Kim and Loh 2001), e.g., CHAID (Kass 1980) and C4.5 (Quinlan 1993). Figure 2 depicts the tree model for carevaluations, a real-world dataset from the UCI Machine Learning repository (Blake and Merz 1998) with $N=1728$ car evaluations divided into $K=4$ classes. This is a dataset with a small number of features, $p=15$, used to predict whether the car evaluation is *unacceptable*, *acceptable*, *good*, or *very good*.

In these greedy heuristic approaches, a criterion is needed to guide the branching at each branch node. In our credit scoring example, at each branch node, one aims to leave (most of) the *good* payers at one branch and (most of) the *bad* payers at the other one. This has been done by optimizing some measure of the purity of a node with respect to the class split in a classification task, e.g., Gini index or entropy, or its homogeneity with respect to the response variable in a regression task, such as, e.g., mean squared error or mean absolute error. Purity, although popular, only measures classification accuracy in an indirect way, and therefore may not yield good generalization results (Fayyad and Irani 1992), and other criteria, such as Maximum Likelihood (Su et al. 2004), have been proposed. At any branch node, one searches for the splitting rule that yields the larger gain in purity or homogeneity of the children versus the parent. For orthogonal cuts, this implies examining the gain for all the predictor variables and all possible values of threshold, as many as individuals in the parent node. Although, in principle, there may be an infinite amount of values for this threshold, just taking the midpoints between consecutive observed values of the predictor variable in the training sample suffices. We refer the reader to Liu et al. (2002) for a comprehensive review on enhancing classification and regression tree methods through the discretization of continuous predictor variables (Dougherty et al. 1995). The process of partitioning finishes when a stopping criterion is satisfied, for instance, when the requirement on the minimum number of individuals at leaf nodes would be violated. Then, a prediction is chosen in each of the leaf nodes. Commonly, for classification, a leaf node is labeled with the most frequent class in the set of individuals that have fallen into the node, while for regression, the prediction equals to the average of the response variable on those individuals, which is the prediction given by a linear model with just an intercept and no predictor variables.

Trees built in this way may still overfit, and therefore, a post-pruning step is performed to remove some unnecessary splits. Pruning is usually performed in a greedy fashion in which leaf nodes are sequentially removed. While, in the forward phase, a purity criterion was considered, now, a criterion combining accuracy and tree complexity is used. The removal of leaf nodes continues, while the value of the criterion improves. See Pangilinan and Janssens (2011) for a bi-objective approach to control both criteria.

To enhance their performance, the greedy procedures were extended in different directions, such as the use of global optimization approaches (Barros et al. 2011; Fu et al. 2003; Grubinger et al. 2014). Below, we will elaborate on two other important generalizations, namely, building trees with oblique cuts or based on combining a collection of trees.

### Building a tree model with oblique cuts

The first enhancement relates to extending orthogonal splits to oblique, a.k.a. multivariate, splits, with implementations such as OC1 (Murthy et al. 1994), oblique.tree (Truong 2009), and HHCART (Wickramarachchi et al. 2016). Trees implementing oblique cuts are more versatile and tend to generate smaller trees with better performance (Brodley and Utgoff 1995; Li et al. 2003). This improvement in accuracy comes with increasing computational times, since the enumerative procedure does not apply anymore and, instead, some sort of optimization problem has to be solved at each branch node. In addition, model interpretability may also be harmed. There have been some proposals to build oblique cuts using a baseline classification method at each branch node, such as Support Vector Machines (Orsenigo and Vercellis 2003) or Logistic Regression (Truong 2009), such that the predictions obtained in this way split the parent node into children. Nevertheless, tackling the optimization of oblique cuts is already at the seminal papers of Bennett (Bennett 1992; Bennett and Blue 1996). For binary classification, she adjusts to the tree context the use of Linear Programming (LP) to build separating hyperplanes (Bennett and Mangasarian 1992). In Bennett (1992), the hyperplane that minimizes the average distance from the misclassified individuals to the hyperplane is modeled as an LP problem, while in Bennett and Blue (1996), for a fixed topology of the tree and fixed predictions at the leaf nodes, the problem of finding the optimal oblique cuts for all branch nodes is written as a set of disjunctive linear inequalities yielding a nonlinear problem. Since these approaches apply only to two-class problems, in Street (2005), multi-class problems are addressed. In Norouzi et al. (2015), and given the challenge of optimizing the empirical loss of the tree model, a convex–concave upper bound is optimized instead, using Stochastic Gradient Descent.

### Building an ensemble of trees

The second enhancement of the strategy discussed in Sect. 2.1 relates to building models that combine the outputs given by a collection of trees, as opposed to a single one, by, for instance, bagging or boosting trees (González et al. 2020). The main exponent of bagging is Random Forests (Biau and Scornet 2016; Breiman 2001; Fawagreh et al. 2014; Genuer et al. 2017), while two of the most popular approaches to boosting are AdaBoost (Freund and Schapire 1997) and Gradient Boosting Machines (Friedman 2001, 2002).

Random Forests (RFs) bag (unpruned) orthogonal trees, and more recently oblique ones (Katuwal et al. 2020; Menze et al. 2011). The trees in the RFs are built independently of each other, on bootstrapped samples of individuals, where the variable selection at each branch node is performed using a random subset of predictor variables. Hence, the trees built differ, because different samples of individuals and features are used. Once the trees are built following the greedy approach described above, RF predicts by combining the predictions of the single trees, e.g., through an average in regression or a majority vote in classification.

AdaBoost is an iterative procedure in which the so-called weak learners (trees of small depth), as well as the individuals, are assigned weights. Individuals for which the prediction was poor in the previous iterations are given a higher weight. Each iteration trains a new weak learner, calculates its error, and defines the weight of the weak learner as well as the weights of the individuals. In some variants of AdaBoost, the weights are optimized (Demiriz et al. 2002; Pfetsch and Pokutta 2020) with techniques such as column generation. While the basic version in classification is designed for two-class classification problems, there are also variants to deal with the multi-class case directly, such as in Hastie et al. (2009), where the authors show that this is equivalent to a forward stage-wise additive modeling algorithm, a.k.a. forward stage-wise boosting, that minimizes a novel exponential loss for multi-class classification.

Gradient Boosting Machines (GBMs) is also an iterative procedure based on combining weak tree learners but usually deeper than in AdaBoost. At the end of each iteration, the residual of the learner at hand is evaluated for each individual. These residuals become the response variable for the next iteration, and therefore, GBM can be seen as a stage-wise additive modeling algorithm. In each iteration, GBM performs a steepest descent minimization for a given loss function, such as mean squared error, mean absolute error, and huber loss functions for regression, and multi-class logistic likelihood for classification. One of the most popular implementations is XGBoost (Chen and Guestrin 2016), which is praised as highly accurate and scalable.

RFs, as well as other methods combining tree models, give, in general, better accuracies than single greedy trees (Fernández-Delgado et al. 2014). However, this is at the expense of losing interpretability and increasing running times. Indeed, these models have a highly complex decision function, being thus less appealing to novel users. The way this lack of interpretability is often addressed is by giving a measure of variable importance, which are often based on permutations of the sample values (Altmann et al. 2010; Louppe et al. 2013; Strobl et al. 2008) or on Game Theory concepts from cooperative games, such as the Shapley value (Casalicchio et al. 2019; Molnar et al. 2018). Recently, there have also been some contributions to enhance model interpretability by replacing the complex model with a simpler surrogate, say a tree model, such that the output of both models is as close as possible. This approach is suggested in Vidal et al. (2020), where the closest tree is extracted using dynamic programming. Alternatively, one can extract a collection of rules, and techniques such as column generation may be used as in Birbil et al. (2020) or heuristics as in Bénard et al. (2019, 2020).

### Shortcomings of the greedy approach

Classic classification and regression trees, as well as the extensions mentioned above, cannot easily include desirable global structural properties, such as model sparsity and cost-sensitivity, due to their greedy nature. Nonetheless, some attempts have been made to address this shortcoming. To enhance model interpretability, one wishes to perform feature selection to control the number of predictor variables used across the tree (Ruggieri 2019). The regularization framework in Deng and Runger (2012) adds to the criterion optimized in each branch node a penalty term for predictor variables that have not appeared yet in the tree, so that the process is reluctant to use too many predictor variables, yielding a sparse tree model. This approach is refined in Deng and Runger (2013), by also including the importance scores of the predictor variables (Louppe et al. 2013; Strobl et al. 2008), obtained in a preprocessing step running a preliminary RF. In the next section, we model sparsity explicitly, and thus, it can be optimized, as we do with the learning performance of the tree model. Similarly, the control on the performance of the tree model in critical/risk groups is done through cost parameters, such as penalizing with a higher cost the errors in the critical groups, as opposed to modeling the corresponding constraints explicitly as we will do in Sect. 4.

## Optimal classification and regression trees

In recent times, and because of the dramatic improvements in hardware and optimization solvers (Bixby 2012), many papers on building optimal (in some sense) classification and regression trees have appeared. In this section, we focus on the Continuous Optimization and the Mixed-Integer Linear Optimization paradigms (Bertsimas and Dunn 2017; Blanquero et al. 2020b; Firat et al. 2020; Günlük et al. 2019). The reader is referred to, e.g., Verhaeghe et al. (2019) for a constraint programming paradigm, an SAT one in Narodytska et al. (2018), Yu et al. (2020), and a dynamic programming one in Demirović et al. (2020). This section aims at comparing the two paradigms in terms of type of decision variables and constraints required to model (1) the movement of individuals along the tree and (2) the prediction rule for new individuals.

### Continuous optimization

In this section, we describe how Optimal Randomized Classification and Regression Trees work and what type of Nonlinear Continuous Optimization formulations have been provided in Blanquero et al. (2021, 2020a, b) to build them. Recall that $\varvec{x}_{i}$ denotes the vector of predictor variables of individual *i*, $i=1,\ldots ,N$. Throughout this section, we typeset other vectors and matrices of decision variables in bold font.

**Fig. 3:**
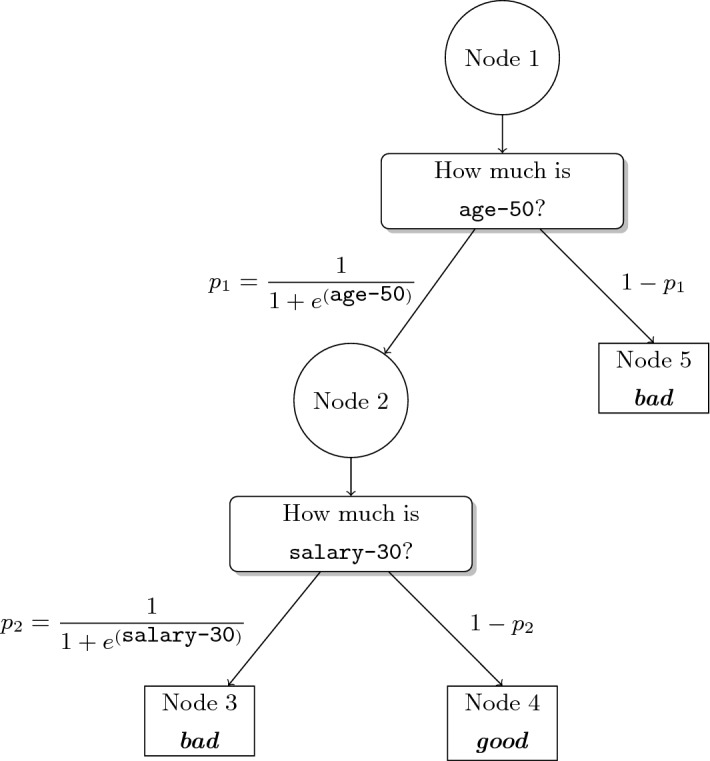
A randomized tree model $\mathcal {T}$ to predict the *good* payers class vs the *bad* payers class, where *F* is the CDF of a logistic random variable

In Optimal Randomized Classification and Regression Trees, the splitting rule at branch node $t \in \tau _B$ is probabilistic (Irsoy et al. 2012; Yang et al. 2018), i.e., individuals move with a certain probability to the left child of *t* and with the complementary probability to the right one. This type of rule is modeled evaluating $F(\cdot )$, the smooth CDF of a univariate continuous random variable, at the splitting rule in node *t*, yielding $p_{it}\left( \varvec{a}_{\cdot t}, \mu _t\right) = F\left( \dfrac{1}{p} \varvec{a}_{\cdot t}^\top \varvec{x}_{i}-\mu _t\right)$. See in Fig. 3 the probabilistic splitting rules at Nodes 1 and 2, where *F* is the CDF of a logistic random variable. With a probabilistic splitting rule, individual *i* moves along all paths in the tree. The probability distribution across the leaf nodes associated with individual *i* is defined by $\{P_{it} \left( \varvec{a}, \varvec{\mu }\right) \}_{t\in \tau _L}$. With the probabilities associated with the individuals and the predictions at the leaf nodes, one can evaluate the total expected error of the randomized tree model. The goal of Optimal Randomized Classification and Regression Trees is to minimize the expected error as well as maximize the so-called local and the global sparsity of the tree. These sparsity terms are modeled with LASSO terms and controlled with their corresponding parameters.

We present below the Continuous Optimization formulation for Optimal Randomized Classification and Regression Trees in Blanquero et al. (2021, 2020a, b) with the purpose of visualizing the conciseness of its feasible region. Before, we need to introduce some notation and decision variables:

*Data*

*D*: depth of the tree,
$$\mathcal {N}^\mathrm{left}_t$$
set of ancestor nodes of leaf node *t* whose left branch takes part in the path from the root node to leaf node *t*, $t\in \tau _L$,
$$\mathcal {N}^\mathrm{right}_t$$
set of ancestor nodes of leaf node *t* whose right branch takes part in the path from the root node to leaf node *t*, $t\in \tau _L$,
$$W_{y_ik} \ge 0$$
misclassification cost incurred when classifying an individual *i*, whose class is $y_i$, in class *k*, $y_i, i=1,\ldots ,N, \,\,k=1,\ldots ,K$,
$$F\left( \cdot \right) $$
smooth CDF of a univariate continuous random variable symmetric w.r.t. 0, used to define the probabilities for an individual to go to the left or the right child node in the tree,
$$\lambda ^\mathrm{local}, \lambda ^\mathrm{global}\ge 0$$
local and global sparsity regularization parameters.

*Decisions*

$$a_{jt}\in \mathbb {R}$$
coefficient of predictor variable *j* in the splitting rule at branch node $t\in \tau _B$, with $\varvec{a}=\left( a_{jt}\right) _{j=1,\ldots ,p,\,\,t\in \tau _B}$. The expressions $\varvec{a}_{j\cdot }$ and $\varvec{a}_{\cdot t}$ will denote the *j*th row and the *t*th column of $\varvec{a}$, respectively,
$$\mu _t\in \mathbb {R}$$
independent term in the splitting rule at branch node $t\in \tau _B$, with $\varvec{\mu }=\left( \mu _t \right) _{t\in \tau _B}$,
$$C_{kt}\ge 0$$
probability of being assigned to class label $k=1,\ldots , K,$ for an individual at leaf node $t,\,\,t\in \tau _L$, with $\varvec{C}=\left( C_{kt}\right) _{k=1,\ldots ,K,\,\,t\in \tau _L}$.

*Probabilities*

$$p_{it}\left( \varvec{a}_{\cdot t}, \mu _t\right) $$
probability of individual *i* going down the left branch at branch node *t*. Its expression is $p_{it}\left( \varvec{a}_{\cdot t}, \mu _t\right) = F\left( \dfrac{1}{p} \varvec{a}_{\cdot t}^\top \varvec{x}_{i}-\mu _t\right)$, $i =1,\ldots , N, \,\, t\in \tau _B$. Note that this probability is a smooth function of $\left( \varvec{a}_{\cdot t}, \mu _t\right)$, due to the smoothness of the CDF *F*.
$$P_{it}\left( \varvec{a}, \varvec{\mu }\right) $$
probability of individual *i* falling into leaf node *t*. Its expression is $P_{it} \left( \varvec{a}, \varvec{\mu }\right) = \prod \limits _{t\in \mathcal {N}^\mathrm{left}_t} p_{it}\left( \varvec{a}_{\cdot t}, \mu _{t}\right) \prod \limits _{t\in \mathcal {N}^\mathrm{right}_t}\left( 1- p_{it}\left( \varvec{a}_{\cdot t}, \mu _{t}\right) \right)$, $i=1,\ldots ,N,\,\, t\in \tau _L,$ and is also a smooth function of $\left( \varvec{a}, \varvec{\mu }\right)$.

With this notation, the Continuous Optimization formulation to build a randomized classification tree model reads as follows:

1 $$\begin{aligned} \underset{(\varvec{a}, \varvec{\mu }, \varvec{C}) \in {\mathcal F}}{\text{ minimize }} \dfrac{1}{N}\sum \limits _{i=1}^N \sum \limits _{t\in \tau _L} P_{it}\left( \varvec{a}, \varvec{\mu }\right) \sum \limits _{k=1}^K W_{y_ik} C_{kt} +\lambda ^\mathrm{local} \sum _{j=1}^p \left\Vert \varvec{a}_{j\cdot }\right\Vert _1 +\lambda ^\mathrm{global} \sum _{j=1}^p \left\Vert \varvec{a}_{j\cdot }\right\Vert _\infty \end{aligned}$$

with

2 $$\begin{aligned} {\mathcal F} = \Bigg\{ (\varvec{a}, \varvec{\mu }, \varvec{C}) \in \mathbb {R}^{p | \tau _B |} \times \mathbb {R}^{| \tau _B |} \times \mathbb {R}^{K | \tau _L |} \, : \, \sum _{k=1}^K C_{kt} =1, C_{kt} \ge 0, \, \forall k=1, \ldots ,K \, \forall t\in \tau _L \Bigg\}. \end{aligned}$$

The objective function of Problem (1) has three terms. The first one is equal to the average misclassification cost in the training sample, while the second and the third ones are regularization terms. The second term addresses the local sparsity of the tree model, penalizing the $\ell _1$-norm of the coefficients of the predictor variables in each of the splitting rules along the tree. The larger the parameter $\lambda ^\mathrm{local}$, the fewer predictor variables in the splitting rules, and thus, the tree model would be more similar to a tree with orthogonal cuts, which is much more interpretable. However, it is also important to control the global sparsity of the tree model, i.e., whether a given predictor variable is ever used by the tree model, and thus, the (hopefully few) predictor variables which really affect the classification/regression are identified. This is done in the third term of the objective function through summing for the different predictor variables the $\ell _\infty$-norm of the vector of coefficients associated with such variable, which forces these coefficients to be shrunk simultaneously across all branch nodes.

**Fig. 4:**
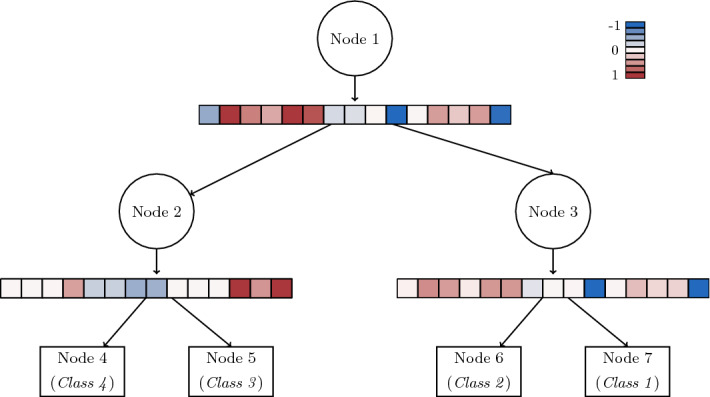
Illustration of Optimal Randomized Classification Tree for carevaluations, with $\lambda ^\mathrm{global}=\lambda ^\mathrm{local}=0$. The classification accuracy of this model is $92.7\%$, while $100\%$ of the predictor variables are used across the tree as well as in each of the three branch nodes. The magnitude of the coefficients of the splitting rule in each branch node is visualized with a heatmap. The heatmap transitions from blue for negative coefficients, to red for positive ones, while white is chosen for values close to 0

**Fig. 5:**
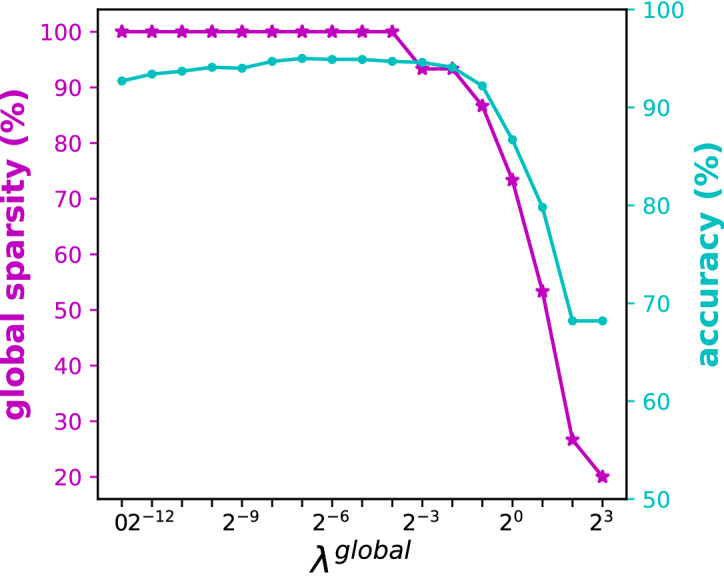
Trade-off between accuracy and global sparsity in Optimal Randomized Classification Trees for carevaluations where $\lambda ^\mathrm{global}$ is varied

In terms of the feasible region $\mathcal {F}$, for each $t \in \tau _L$, we need to impose semi-assignment constraints as well as nonnegativity of $C_{kt}$ to ensure that $\{C_{kt}\}_{k=1}^K$ is a probability distribution for the class assignment in leaf node $t\in \tau _L$. Note that, since the technology matrix satisfies the total unimodularity property and the objective function is linear for fixed $(\varvec{a}, \varvec{\mu })$, there exists an optimal solution to Problem (1), such that $C_{kt} \in \{0,1\}$, meaning that each leaf node predicts exactly one class. Figure 4 plots the Optimal Randomized Classification Tree for carevaluations, with $\lambda ^\mathrm{global}=\lambda ^\mathrm{local}=0$, while Fig. 5 illustrates the trade-off between accuracy and global sparsity when $\lambda ^\mathrm{global}$ is varied.

Once the tree model is built, the prediction of future data is done as follows. Let $(\varvec{a}^*,\varvec{\mu }^*,\varvec{C}^*)$ be the optimal solution to Problem (1). The probability of individual $i \in \mathcal {I}$ being assigned to class *k* is equal to $\sum _{t\in \tau _L} P_{it}\left( \varvec{a}^*,\varvec{\mu }^*\right) C^*_{kt}$, for each $k=1,\ldots ,K$. For an incoming individual with predictor vector $\mathbf {x}$, the probability of belonging to class *k* returned by the randomized tree is equal to:

3 $$\begin{aligned} \mathbf {x} \rightarrow \Pi _k(\mathbf {x}):=\sum _{t\in \tau _L} P_{\mathbf {x} \, t}\left( \varvec{a}^*,\varvec{\mu }^*\right) C^*_{kt}, \end{aligned}$$

where $P_{\mathbf {x} \, t}\left( \cdot ,\cdot \right)$ is defined similarly to $P_{i t}\left( \cdot ,\cdot \right)$ where $\mathbf {x}$ replaces $\mathbf {x}_i$. Note that $\Pi _k(\cdot )$ is smooth in the continuous predictor variables, since the CDF *F* is assumed to be a smooth function. This means that even small changes in these variables will produce changes in $\Pi _k(\cdot )$. This is not the case for deterministic tree models such as CART and RF, where there are no changes at all in the class membership probabilities when there are small changes in the continuous predictor variables. The output associated with each $\mathbf {x}$ is probabilistic, namely, the vector of probabilities $\left( \Pi _1(\mathbf {x}), \ldots , \Pi _K(\mathbf {x})\right)$. If a deterministic classification is sought, the class predicted for $\mathbf {x}$ is $k(\mathbf {x}) \in \arg \max \{\Pi _k(\mathbf {x}), k=1,\ldots ,K\}$.

Problem (1) has $(p+1) |\tau _B|$ + $K |\tau _L|$ continuous decision variables, associated with the coefficients of the predictor variables, including the independent terms, as well as with the class assignment, and $|\tau _L|$ linear constraints relating to the class assignment too. The first term in the objective function is smooth, while the other two terms are not, due to the $\ell _1$ and $\ell _\infty$ norms. With standard techniques, we can find an equivalent smooth formulation, which can be given to any nonlinear solver that can deal with constrained problems. The number of nodes in the tree, and thus the number of decision variables in Problem (1), grows exponentially with the depth of the tree, *D*. Hence, solving Problem (1) may be time demanding for large or even moderate values of *D*. Fortunately, the computational experiments in Blanquero et al. (2021, 2020a, b) illustrate that good accuracies can be achieved with small values of *D*, namely, $D \le 4$.

Several important remarks on Problem (1) follow. First, the feasible region ${\mathcal F}$ in (2) speaks favorably toward the scalability of Problem (1) with respect to the size of the training sample. Indeed, when the number of individuals *N* grows, the feasible region remains of the same size, since there are no decision variables directly relating to the individuals. Hence, although the evaluation of the objective function becomes more time demanding with larger *N*, the dimensionality of the problem to be solved remains the same. Second, there are two regularization terms that can help with feature selection, i.e., identify a subset of predictor variables with a good trade-off between accuracy and sparsity. Third, we can perform with standard techniques a full sensitivity analysis to study the impact that predictor variables have on the class prediction for each individual. Recall that the function $\Pi _k(\cdot )$ in (3) is smooth in the continuous predictor variables. Therefore, we have that small changes in the continuous predictor variables in a given individual lead to small changes in the values of the probabilities of class membership, since $\Pi _k(\cdot )$ can be approximated by its first order Taylor expansion. This means that, for any individual, we can perform a full sensitivity analysis to study the impact that each continuous predictor variable has on the probability of class membership. This is a step forward toward local explainability of tree models, addressed in Sect. 4.

To end this section, we note that Problem (1) can easily be modified for regression. Indeed, one needs to replace the information relating to the prediction of the *K* classes and the loss incurred, by the prediction of the (continuous) response at each leaf node and a suitable loss. In terms of prediction, one can use any regression model that is compatible with an optimization approach to learning, such as, e.g., a linear, a generalized linear, or an LASSO model. For each individual, the prediction is the expected value of the predictions made at the different leaf nodes, using the probability distribution $\{P_{it} \left( \varvec{a}, \varvec{\mu }\right) \}_{t\in \tau _L}$. If we take, for instance, the mean squared error, we would have the following unconstrained problem:

$$\begin{aligned} \underset{(\varvec{a},\varvec{\mu },\varvec{\tilde{a}},\varvec{\tilde{\mu }}) \in \mathbb {R}^{(p+1) (| \tau _B |+| \tau _L |)}}{\text{ minimize }} \qquad \dfrac{1}{N}\sum \limits _{i=1}^N \Big (\sum \limits _{t\in \tau _L} P_{it}\left( \varvec{a}, \varvec{\mu }\right) \, (\varvec{\tilde{a}}_{\cdot t}^\top \varvec{x}_{i}+\tilde{\mu }_t ) - y_i \Big )^2 +\lambda ^\mathrm{local} \sum _{j=1}^p \left\Vert (\varvec{a}_{j\cdot },\tilde{\varvec{a}}_{j\cdot })\right\Vert _1 +\lambda ^\mathrm{global} \sum _{j=1}^p \left\Vert (\varvec{a}_{j\cdot },\tilde{\varvec{a}}_{j\cdot })\right\Vert _\infty . \end{aligned}$$

As for Problem (1), and by rewriting the regularization terms, this can be reformulated as a smooth problem with linear constraints. Other losses can be easily modeled too, such as the mean absolute error or the quantile regression (Koenker and Hallock 2001; Kriegler and Berk 2010). These losses though need to be rewritten, in a similar fashion as for regularization terms, to ensure the smoothness of the objective function. For these losses, none of the decision variables is directly associated with the individuals, and therefore, the dimension of the optimization problem behind regression still does not depend on the size of the training sample *N*.

### Mixed-integer linear optimization

In this section, we review Mixed-Integer Linear Optimization (MILO) approaches to build Optimal Classification and Regression Trees. The key issue in this paradigm is that one controls the path each individual takes and thus calls for the modeling of (many) binary decision variables. We start with the approach in Bertsimas and Dunn (2017), Dunn (2018), and discuss how it compares to the continuous formulation in the previous section. We then continue by reviewing other relevant literature, which involves different decision variables and/or more sophisticated solution approaches.

In Bertsimas and Dunn (2017), the aim is to build a deterministic optimal binary tree of depth *D* guided by two objectives, namely, the misclassification error and the complexity of the tree, where the latter is measured as the summation across all branch nodes of the number of predictor variables used in the splitting rules. The MILO formulation in Bertsimas and Dunn (2017), OCT-H in Eq. (28) on pages 1054–1055, requires the following notation and decision variables:

*Data*

*D*: depth of the tree,
$$\alpha \ge 0$$
complexity parameter in the objective function,

*Decisions*

$$d_{t}\in \left\{ 0,1\right\} $$
1 if a cut is applied at branch node *t*, $t\in \tau _B$,
$$l_{t}\in \left\{ 0,1\right\} $$
1 if leaf node *t* contains individuals, $t\in \tau _L$,
$$a_{jt}\in \left[ -1,1\right] $$
coefficient of predictor variable *j* in the splitting rule at branch node *t*, $j=1,\ldots ,p, \,\, t\in \tau _B$,
$$\mu _t\in \mathbb {R}$$
independent term in the splitting rule at branch node *t*, $t\in \tau _B$,
$$z_{it}\in \left\{ 0,1\right\} $$
1 if individual *i* is in leaf node *t*, $i=1,\ldots ,N, \,\, t\in \tau _L$,
$$C_{kt}\in \left\{ 0,1\right\} $$
1 if leaf node *t* is labeled with class $k,\,\, k = 1,\ldots , K,\,\, t\in \tau _L$,
$$s_{jt}\in \left\{ 0,1\right\} $$
1 if predictor variable *j* is used at branch node $t,\,\, j=1,\ldots ,p,\,\, t\in \tau _B$.

As in Sect. 3.1, $\varvec{d}$, $\varvec{l}$, $\varvec{a}$, $\varvec{\mu }$, $\varvec{z}$, $\varvec{C}$, and $\varvec{s}$ denote the corresponding vector/matrix of decision variables. This formulation requires binary decision variables to define the topology of the tree, namely, $\varvec{d}$ and $\varvec{l}$; continuous decision variables $\varvec{a}$ and $\varvec{\mu }$ to define the splitting rules; binary decision variables $\varvec{z}$ to control in which leaf node the individuals are placed by the tree model; binary decision variables $\varvec{C}$ associated with the class prediction; and binary decision variables $\varvec{s}$ to control the local sparsity of the tree model. This formulation requires more decision variables than Problem (1), many of them are binary, and the number of some of them, $\varvec{z}$, linearly depends on the size of the training sample *N*. Therefore, as noted in Dunn (2018), this approach is only feasible for moderate values of *N*.

With regards to the objective function, it consists of two terms, and their linear combination through parameter $\alpha$ is to be minimized. The first term in the objective function represents the total misclassification error across all leaf nodes, assuming that individuals are assigned to the majority class in the leaf node they have been assigned to. Auxiliary decision variables are required to linearize the maximization in the majority class as well as big-*M* constraints linking them with variables $C_{kt}$. Thus, we cannot use the same arguments as in the previous section, to prove that the integrality constraints of $C_{kt}$ can be relaxed without loss of optimality. The second term of the objective function measures the local sparsity of the tree, by counting the number of predictor variables used at each branch node and summing these up, which can be done through decision variables $\varvec{s}$. This formulation has a nonlinear objective function as in Problem (1), but as mentioned above, one can define additional variables and additional constraints to linearize it. In addition, the regularization terms in Problem (1) are replaced by a term that fully controls the local sparsity with binary decision variables. Global sparsity, although not modeled in Bertsimas and Dunn (2017), can be included, e.g., with an LASSO term as in Sect. 3.1, or by adding new binary decision variables and additional constraints, linking them to the existing ones $\varvec{s}$.

In terms of the feasible region, and as in Problem (1), we have the semi-assignment constraints associated with $\varvec{C}$. In addition, there are other constraints that need to be included in the MILO formulation. Indeed, since this approach is deterministic, we need to impose that each individual reaches exactly one leaf node, and that $\varvec{z}$ are well-defined, i.e., they are compatible with splitting rules applied at the branch nodes. There are well-defined constraints between $\varvec{s}$ and $\varvec{d}$. We have big-*M* constraints to ensure that $a_{jt}=0$ if predictor variable *j* is not used in branch node *t*, i.e., $s_{jt}=0$. There are also big-*M* constraints to ensure that $z_{it}=0$ if node *t* does not contain individuals, i.e., $l_{t}=0$, as well as to force that the corresponding coefficients $\varvec{a}$ and $\varvec{\mu }$ to be zero if no split is applied at a branch node. Finally, we have to forbid that a branch node splits if its parent did not, except for the root node.

This formulation can be given to any MILO solver. As in Sect. 3.1, the computational experiments in Bertsimas and Dunn (2017) illustrate that good accuracies can be achieved with small values of *D*, but at a considerable computational cost for small and medium problem instances. To reduce this computational burden, a local search approach is proposed in Dunn (2018), where the MILO formulation is solved for the subproblems associated with branch nodes, thus yielding smaller formulations that are solved repeatedly. With this local search procedure, it is possible to deal with deeper trees, $D \le 10$, more efficiently. However, it is harder to directly control, for instance, the global sparsity of the tree, a crucial issue if, on the top of having a procedure yielding high accuracies, identification of the relevant predictor variables is sought. Moreover, contrary to the randomized trees, we cannot perform a proper sensitivity analysis to explain how small perturbations on a given feature affect the prediction. This means that it is not easy to identify the relevant variables for a given individual, while this is obtained as a byproduct for randomized trees.

The MILO formulation we have just described can be modified to implement orthogonal cuts by making $\varvec{a}$ binary, while the feasible region and the objective function require some small changes. The formulation can also be modified to address a regression task (Dunn 2018), as we have done for Problem (1).

There are other approaches in the literature within the MILO paradigm. In Aghaei et al. (2020), a flow-based MILO formulation is proposed for binary predictor variables. A sink node is added to the tree, yielding a directed acyclic graph (Ahuja et al. 1993). Only individuals ending up in the sink are correctly classified, while flow conservation constraints are imposed at the other nodes of the tree. In Firat et al. (2020), an alternative formulation is proposed with new decisions variables associated with the paths from the root node to the leaf nodes and their splits, which is solved with a column generation-based heuristic. In Günlük et al. (2019), an MILO formulation for combinatorial splitting rules for categorical variables is proposed, i.e., rules defined by a subset of categories that move individuals to the left child node if the rule is satisfied and to the right child node, otherwise, yielding a binary representation of them (Carrizosa et al. 2019).

With the MILO approach, we face the curse of dimensionality, since the number of binary decision variables grows linearly with the size of the training sample *N*. Recent attempts to address this can be found in the literature. An alternative formulation is proposed in Verwer and Zhang (2017), Verwer et al. (2017, 2019) with a more compact feasible region that aggregates some of the constraints described above. In Zhu et al. (2020), a subset of the training sample is selected in a preprocessing step using an LP problem, while, in Zantedeschi et al. (2020), a continuous relaxation is developed.

## Challenges for the future

Throughout this paper, we have illustrated how powerful optimization is to construct classification and regression tree models $\mathcal {T}$ that show a good trade-off between accuracy and sparsity. This section is devoted to discuss new challenges posed by desirable properties we may want to seek or by the complexity of the data at hand, and the first steps that the Mathematical Optimization community has taken to give answers to them. The first research avenue we touch on consists of expanding the family of criteria under consideration: on the top of accuracy and sparsity, there may be other important requirements on $\mathcal {T}$, such as fairness, to ensure that $\mathcal {T}$ protects sensitive groups (Romei and Ruggieri 2014), or explainability, to ensure that the knowledge gained is actionable (Aouad et al. 2019; Bertsimas et al. 2019; Cui et al. 2015; Höppner et al. 2020) in, for instance, the design of drug therapies (Mišić 2020). The second research avenue consists of designing tree models for more complex data: we will discuss the new challenges that arise when some of the predictor variables available to construct $\mathcal {T}$, or even the response, may not be continuous or categorical, and novel tree models are required with these new types of data. Although of interest, this section does not address the asymptotic behavior of tree models: assuming data to be a random sample from a given distribution, an important question is to identify the statistical convergence of the random sequence of optimal trees and optimal values (e.g., optimal expected squared error in a regression tree) when the size of the training set goes to infinity. Very limited results are available in the literature, making strong assumptions on the structure of the tree models. The reader is referred to Biau et al. (2008), Denil et al. (2013), Scornet (2016), and Scornet et al. (2015) for some results in this line.

While there has been a paramount increase in the use of Machine Learning in Decision-Making, it is less well understood how models arrive to decisions. Yet, transparent (a.k.a. interpretable, comprehensible, understandable) models, (Cerquitelli et al. 2017; Hofman et al. 2017), are desirable in Medical Diagnosis (Freitas 2014; Ustun and Rudin 2016), or Criminal Justice (Jung et al. 2017, 2020), to name a few. While a black-box model could be extremely good at predicting who would benefit from a policy intervention, policy makers should be able to explain why decisions are taken, as is evident, e.g., in the COVID-19 crisis. Moreover, transparency (Chen et al. 2017) is a must when, for instance, benchmarking the providers of utilities (Benítez-Peña et al. 2020a) or in credit scoring in consumer lending (Baesens et al. 2003), the reason being the so-called right-to-explanation in algorithmic decision-making, imposed by the European Union since 2018 (European Commission 2020; Goodman and Flaxman 2017; Wachter et al. 2017). Although the term Explainable Artificial Intelligence (*XAI*) was coined a while ago, it is now tracking a lot of attention from different communities, see, e.g., Barredo Arrieta et al. (2020), Gunning and Aha (2019), Holter et al. (2018), Miller (2019).

There is a big body of literature relating to a common surrogate for explainability, namely, model sparsity. The aim there is to perform feature selection to work with a smaller number of predictor variables as a first step toward explaining the behavior of the model globally. This can be done after the model has been built, through variable importance measures, deleting those variables with a small importance (Cohen et al. 2007; Guyon and Elisseeff 2003). Examples of these measures were given in Sect. 2, and are based, for instance, on calculating the impact on accuracy by permuting the values of the feature under investigation, or, inspired by cooperative games, calculating the contribution toward the accuracy of the feature to any coalition of features. Alternatively, we can embed the sparsity in the optimization model solved to train the model, as we have seen in the optimal trees reviewed in the previous section, either with LASSO terms (Hastie et al. 2015) or zero-norm terms (Weston et al. 2003).

Equally important is to give local explanations, i.e., at the individual level, say $\varvec{x}^{0}$. Take, for instance, the stylized credit scoring tree in Fig. 1 and the customer discussed in the introduction of age 43 and salary 28. Recall this individual was assigned to the *bad* payers class, and therefore was denied the credit. The tree model in Fig. 1 has made this decision, because age is below 50 and salary is also below 30. This explanation, however, is of limited use to the individual who would still not understand how to improve the credit score to be labeled as *good*, and thus get the credit granted. Moreover, this type of explanations, i.e., the ones given by the path from the root to the corresponding leaf node, can be arbitrarily long (Izza et al. 2020). Instead, one can offer to individuals counterfactual explanations (Fernández et al. 2020; Lucic et al. 2020; Mothilal et al. 2020). See Karimi et al. (2020), Sokol and Flach (2019), Verma et al. (2020) for recent surveys on counterfactual explanations. This is an explanation about how features need to change to obtain a different class prediction. For our individual at hand, with $\varvec{x}^{0}=(43,28)$, we can say that we have labeled him/her as *bad*, but that with the same age and salary above 30, the label would have been *good* instead. This is an example of *local explainability*, (Molnar et al. 2020), in which the aim is to identify a small set of features and their corresponding value to make the prediction change.

The goal of local explainability is, therefore, to understand which are the predictor variables that have the largest impact on the individual prediction. In case of a linear regression model, this analysis can naturally be performed using the coefficients $\beta _j$, $j=1,\ldots ,p$, of the predictor variables. If variable *j* changes by $\Delta _j$ units, then the response variable changes by $\beta _j \Delta _j$ units, which is clearly independent of the individual. For nonlinear models, one can make use of model-agnostic approaches to build local explanations, such as the so-called Local Interpretable Model-agnostic Explanations (LIME) (Ribeiro et al. 2016). The latter relies on building linear approximations to the model at $\varvec{x}^{0}$, using predictor vectors generated in the neighborhood of $\varvec{x}^{0}$ and predictions obtained with the original model. Although popular, there are a number of shortcomings to this approach around, for instance, the generation of data or the loss of accuracy, which have been addressed with enhancements (Botari et al. 2020; Visani et al. 2020; Zhang et al. 2019), or with alternative approaches (Lundberg et al. 2020, 2018; Lundberg and Lee 2017).

Instead, and as advocated by Rudin (2019), it is better to work with models that can derive local explanations directly, as is the case for Neural Networks (Gevrey et al. 2003) but also for the Optimal Randomized Classification and Regression Trees in Sect. 3.1. To illustrate this, and for simplicity, we consider a classification problem where all predictor variables are continuous. For an individual with predictor variables $\varvec{x}^{0}$, we analyze how an infinitesimal change $\varvec{\Delta }\in \mathbb {R}^p$ in the predictor variables affects the probability $\Pi _{k}$ of being in class $k, k=1,\ldots ,K$. By linearizing $\Pi _{k}$ close to $\varvec{x}^{0}$, we have:

$$\begin{aligned} \Pi _{k} (\varvec{x}^{0}+\varvec{\Delta }) \approx \Pi _{k} (\varvec{x}^{0})+ \sum _{j=1}^p\frac{ \partial \Pi _{k} }{\partial x_j} (\varvec{x}^{0}) \cdot \Delta _j. \end{aligned}$$

Thus, the matrix of partial derivatives

$$\begin{aligned} \left( \frac{ \partial \Pi _{k} }{\partial x_j} (\varvec{x}^{0}) \right) _{\begin{array}{c} k=1,\ldots K\\ j=1,\ldots ,p \end{array}} \end{aligned}$$

gives full information on the sensitivity of the class membership probabilities $\Pi _{k}$ around $\varvec{x}^{0}$.

Even more, we can provide counterfactual explanations to an individual with $\varvec{x}=\varvec{x}^{0}$ on what are the minimum changes to the predictor variables, such that the individual with $\varvec{x}=\varvec{x}^{0}+\varvec{\Delta }$ is predicted to be in class $k^*$. Indeed, given a norm $\Vert \cdot \Vert$ and a set $\mathcal {A} \subset \mathbb {R}^p$ of allowed movements from $\varvec{x}^{0}$, one can solve a nonlinear problem of the form:

$$\begin{aligned} \begin{array}{rll} \text{ minimize}_{\varvec{\Delta }} &{} \Vert \varvec{\Delta }\Vert &{} \\ s.t. &{} \Pi _{k^*} (\varvec{x}^{0}+\varvec{\Delta }) \ge \Pi _{k} (\varvec{x}^{0}+\varvec{\Delta }) &{} \forall k =1,\ldots ,K\\ &{} \varvec{\Delta } \in \mathcal {A}. &{} \end{array} \end{aligned}$$

The use of Machine Learning models in socially sensitive decision-making calls for analyzing their fairness (Iosifidis and Ntoutsi 2019; Miron et al. 2020; Zafar et al. 2017). The fairness can refer to the accuracy achieved in risk groups, for which the consequences of a wrong prediction are much more severe than for the rest (Kao and Tang 2014; Turney 1995). These examples abound, for instance, in medical diagnosis. The most natural way to handle this cost-sensitivity is to add the so-called performance constraints, one for each risk group, to ensure that the accuracy achieved in an independent sample is acceptable, i.e., above a threshold. Second, fairness can also refer to avoiding that the outcome of the model discriminates groups of people sharing sensitive features, such as gender or race, (Miron et al. 2020). This has gained attention due to the increase of automatization in decision-making, but also concerns that existing biases in data may be amplified by, not carefully built, data-driven tools.

The goal is to build a model with high accuracy, but at the same time prevent any type of discrimination, either direct because it uses sensitive data, or indirect because the prediction is disproportionally negatively impacted in those individuals, although no sensitive features have been used. New criteria have been defined to achieve this, namely disparate treatment and disparate impact (Barocas and Selbst 2016; Zafar et al. 2017), and in recent works, optimal trees have been extended to include them (Aghaei et al. 2019). The extension of the models in Sect. 3.1 to address this issue is straightforward. To illustrate this, and again wlog, we consider a classification problem. Suppose that we have a group $\mathcal {S} \subset \{1,\ldots ,N\}$ of individuals to be protected against discrimination by Problem (1). We may impose that the average probability of being assigned to class *k* for individuals in $\mathcal {S}$ does not differ much from the average in the whole training sample $\mathcal {I}$. This can be model through the following constraint:

$$\begin{aligned} \left| \frac{1}{|\mathcal {S}|} \sum_{i\in \mathcal {S}} 
\sum_{t\in\tau_L} P_{it} \left(\varvec{a},\varvec{\mu}\right)C_{kt} - \frac{1}{N} \sum_{i=1}^N\sum_{t\in\tau_L} P_{it}\left(\varvec{a},\varvec{\mu}\right)C_{kt}\right| \le \varepsilon , \end{aligned}$$

for $\varepsilon >0$ sufficiently small.

We end the section with some considerations on other types of data, apart from continuous and binary, available to construct tree models that show a good trade-off between accuracy and sparsity. When building classification and regression models, there may be characteristics which are recorded as, for example, time-series data (Barrow and Crone 2016; Carrizosa et al. 2013; Saha et al. 2020), spatial data (Georganos et al. 2019), functional data (Balakrishnan and Madigan 2006; Möller et al. 2016; Pospisil and Lee 2019; Rahman et al. 2019), text data (Martens and Provost 2014; Ramon et al. 2020), or network data (Óskarsdóttir et al. 2020), which are not captured appropriately by standard implementations of these models. This calls for new mathematical optimization formulations and/or numerical solution approaches to address these complexities adequately. The changes may stem from the functions we use to measure accuracy or sparsity. The typical losses such as the mean squared error or the expected misclassification cost may not be suitable to measure the accuracy for more complex response variables. In terms of sparsity, take, for instance, the case of time-series data, where we have an observation for each time period in the series, the response for this observation is the measurement in that time period and the features are the measurements in previous time periods, as in Benítez-Peña et al. (2020b) for the short-term predictions of the evolution of COVID-19. In this way, we have that individuals are characterized by *p* lags, but possibly other predictor variables. In addition to the ones described in Sect. 3, one may wish other types of sparsity, ensuring that only recent lags are used, or that as few as possible series are used in multivariate time-series (Tuncel and Baydogan 2018), or even more sophisticated versions of sparsity for hierarchical ones (Athanasopoulos et al. 2017; Karmy and Maldonado 2019; Wickramasuriya et al. 2019). For classification and regression problems with functional data, i.e., functions $x_i: [0,1] \rightarrow \mathbb {R},$ one can easily adapt the models in Sect. 3.1 by replacing the definition of the probabilities $p_{it}\left( \varvec{a}_{\cdot t}, \mu _t\right)$ at branch node *t* by, for instance:

$$\begin{aligned} F\left( \gamma _{1t} \int _{0}^{c_1} x_i(s) \, d s + \gamma _{2t} \int _{c_1}^{c_2} x_i(s) \, d s + \ldots \gamma _{r t} \int _{c_{r-1}}^{1} x_i(s) \, d s\right) , \end{aligned}$$

where the thresholds $c_1,c_2,\ldots ,c_{r-1}$ and the weights $\gamma _{1t}, \gamma _{2t},\ldots , \gamma _{rt}$ are decision variables. See, for instance, (Blanquero et al. 2019, 2020) for a related approach in Support Vector Machines. The examples above show that new forms of losses and/or sparsity can be incorporated in both the Continuous Optimization and the MILO paradigms, by making changes to the objective function, but new decisions as well as new constraints may be required, yielding challenging Mixed-Integer Nonlinear Optimization formulations.

## Conclusions

The impressive advances in hardware and software in the last decades have allowed the development of more powerful versions of classification and regression trees than classic ones. In this paper, we have reviewed recent Continuous Optimization and Mixed-Integer Linear Optimization formulations to build optimal classification and regression trees that trade off accuracy and sparsity, the latter understood as a proxy for interpretability. Contrary to standard classification and regression trees built in a greedy heuristic manner, formulating the design of the tree model as an optimization problem allows the inclusion, either as hard or soft constraints, of other important criteria. We have illustrated this flexibility for an important social criterion, the fairness of the model, which aims to avoid predictions that discriminate against race, or other sensitive data, and/or ensures an acceptable accuracy performance for groups at risk. We have also shown how optimization provides in a natural way counterfactual explanations for individuals, enhancing the local explainability of tree models. In the future, we foresee new optimization models which will be needed to tailor optimal trees to complex data arising in decision-making, yielding large-scale global optimization problems, usually with integer variables, and sophisticated numerical optimization strategies are to be devised to address these challenging problems.
